# A Review of the Recent Theories of Nerve Action, the Use of Electricity in Medicine and Surgery, &c.

**Published:** 1878-02

**Authors:** 


*A Review of the Recent Theories of Nerve Action, the* Use *of Electricity in Medicine and Surgery, &c.*—By John J. Caldwell, M. D., Baltimore, Md, 1877.

This is a reprint of several articles which have appeared in this and other Journals concerning the use of electricity and its effects upon Brain and Nerve Matter, proving a very interesting pamphlet of some twenty pages.

